# Stability of Two-Dimensional Liquid Foams under Externally Applied Electric Fields

**DOI:** 10.1021/acs.langmuir.2c00026

**Published:** 2022-05-12

**Authors:** Matthieu Fauvel, Anna Trybala, Dmitri Tseluiko, Victor Mikhilovich Starov, Himiyage Chaminda Hemaka Bandulasena

**Affiliations:** † Department of Chemical Engineering, Loughborough University, Loughborough, Leicestershire, LE11 3TU, United Kingdom; ‡ Department of Mathematics, Loughborough University, Loughborough, Leicestershire, LE11 3TU, United Kingdom

## Abstract

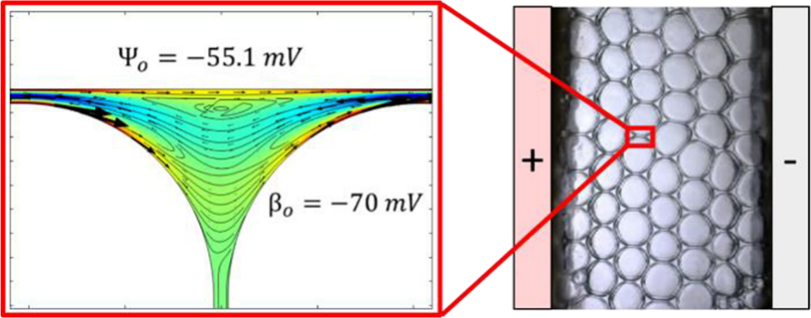

Liquid foams are highly complex systems consisting of gas bubbles trapped within a solution of surfactant. Electroosmotic effects may be employed to induce fluid flows within the foam structure and impact its stability. The impact of external electric fields on the stability of a horizontally oriented monolayer of foam (2D foam) composed of anionic, cationic, non-ionic, and zwitterionic surfactants was investigated, probing the effects of changing the gas–liquid and solid–liquid interfaces. Time-lapse recordings were analyzed to investigate the evolution of foam over time subject to varying electric field strengths. Numerical simulations of electroosmotic flow of the same system were performed using the finite element method. Foam stability was affected by the presence of an external electric field in all cases and depended on the surfactant type, strength of the electric field, and the solid material used to construct the foam cell. For the myristyltrimethylammonium bromide (MTAB) foam in a glass cell, the time to collapse 50% of the foam was increased from ∼25 min under no electric field to ∼85 min under an electric field strength of 2000 V/m. In comparison, all other surfactants trialed exhibited faster foam collapse under external electric fields. Numerical simulations provided insight as to how different zeta potentials at the gas–liquid and solid–liquid interfaces affect fluid flow in different elements of the foam structure under external electric fields, leading to a more stable or unstable foam.

## Introduction

Liquid foams are a multiphase medium consisting of gas bubbles dispersed through a continuous liquid phase, with a wide range of applications, such as enhanced oil recovery, food, firefighting, mining, and pharmaceuticals. Novel electrophoretic separation techniques using liquid foams have been proposed recently.^1^ At the nano-scale, the coupling of physical forces may allow unique separation techniques to be developed, considering the fact that the motion of macromolecules is complex as steric interactions and polarization effects come into play.^2^ This presents an opportunity to develop novel foam separation techniques, exploiting the high surface area available in foam and unique analyte dynamics in nano/microchannels to perform efficient separations that are not possible at the macro-scale^3−6^ or using nanochannels acting as filters during electrophoretic separation.^7^ Liquid foams may be described as a network of micro- and nanochannels that can be produced or collapsed on demand in a short period of time with minimal effort and may form a suitable platform for novel separation techniques. A key drawback of most liquid foams in ambient conditions is their uncontrollable lifetime, as they gradually collapse over time due to liquid drainage, and subsequent collapse of the gas bubbles. The ability to exert control over the foam structure and its lifespan is beneficial in manufacturing aerated materials, for instance, building and insulation,^8^ and more recently rechargeable batteries.^9^

Electrokinetic effects may provide a mechanism to control the lifespan of a foam by inducing fluid flows toward or opposite the direction to gravity drainage.^10,11^ Electroosmotic flow can be induced on charged surfaces, for example, at the interface of an ionic-surfactant-stabilized foam, by applying an external electric field across the foam. Surfactants adsorbed onto interfaces could change surface properties significantly by lowering the surface tension and affecting the charge at the interface. The surface charge will attract counterions from the bulk liquid to the interface, forming a thin layer of charged ions at the vicinity of the interface, known as the electrical double layer (EDL). When an external electric field is applied, charged ions in the EDL can be transported by the electric field tangential to the interface, generating a fluid flow. The effects of electroosmotic flows on surfactant-stabilized interfaces have been investigated for surfactant-laden freely suspended films and simple soap films.^10,12,13^ The effects of gravity and capillary driven drainage in liquid foams are well documented;^14−16^ foam drainage equations have been derived from mass balances and liquid momentum equations^17−19^ and numerically solved for multiple situations.^20^ The electrokinetic transport in the vicinity of solid–liquid interfaces is well known,^2^ but electrokinetic effects near gas–liquid interfaces are not completely understood.^10^ The effects of pH^21^ and thermal gradients^22^ have been investigated by few groups, but the effects of different types of surfactants on gas–liquid interfaces, and subsequent foam stability under electrokinetic flow, are poorly understood. Sett et al.^11^ studied the stability of a single soap bubble under an external electric field applied vertically and reported increases in stability for both cationic and anionic surfactants irrespective of the direction of the electric field. Bonhomme et al.^10^ demonstrated the applicability of electrokinetic flow to reverse the drainage of liquid in foam yet noted a period of spontaneous collapse despite the initial increase in stability. It was also found that foam collapse can be accelerated by increasing the electric field strength. These contradictory results reported for free liquid film experiments warrant a systematic investigation of foam stability under external electric fields, especially for different types of surfactants.

The main purpose of this study was to investigate the effects of electrokinetic flow on the stability of a monolayer of liquid foam stabilized by different types of surfactants. Electroosmotic flow depends on the surface properties of the interface, most notably the zeta potential, ζ,^23^ which can be altered by changing the type of surfactant used to stabilize the foam. In this paper, the lifetimes of foams prepared with multiple liquid formulations were examined under varying applied electric fields, demonstrating the effects of electrokinetic flows on anionic, cationic, non-ionic, and zwitterionic surfactants. By fabricating a thin (2 mm) foam cell to contain a monolayer of foam, a method of assessing foam stability over time was developed, aiming to study a consistently repeatable foam structure, in which the effects of gravity drainage may be reasonably ignored to isolate the impact of electrokinetic flow. This repeatable foam structure was used to investigate liquid foams prepared by various surfactant types and how the stability of each foam formulation responds to electrokinetic effects. Experimental results are compared and analyzed with numerical simulations to understand changes in stability for each case.

## Materials and Methods

### Solution Preparation

The test solutions were prepared by mixing 25 g of Milli-Q water (15 MΩ·cm deionized water) with 25 g of glycerol (Sigma-Aldrich, U.K.). Glycerol was added to the solution to increase viscosity and reduce foam drainage. This water–glycerol mixture was used to prepare four separate solutions containing the surfactants SDS (anionic), MTAB (cationic), Triton X-100 (non-ionic), and SB3-14 (zwitterionic) at 1 critical micelle concentration (CMC). All surfactants were purchased from Sigma-Aldrich, U.K. The concentration of surfactants in each solution is presented in Table 1. The viscosities of all solutions were measured to be (4.01 ± 0.05) × 10^–3^ Pa·s, i.e., not affected by the addition of the surfactant at 1 CMC. The pH of all solutions was measured to be 6.5 ± 0.1.

**Table 1:** List of Surfactants Used in the Experiments, Their Type, and CMC^a^

name	surfactant type	CMC
sodium docecyl sulfate (SDS)	anionic	8.2 mM^24^
myristyltrimethylammonium bromide (MTAB)	cationic	4–5 mM^25^
Triton X-100	non-ionic	0.24 mM^26^
myristyl sulfobetaine (SB3-14)	zwitterionic	0.4 mM^27^

a Chemical structures included in Supporting Information Figure S1.

### Experimental Setup

**Figure 1:**
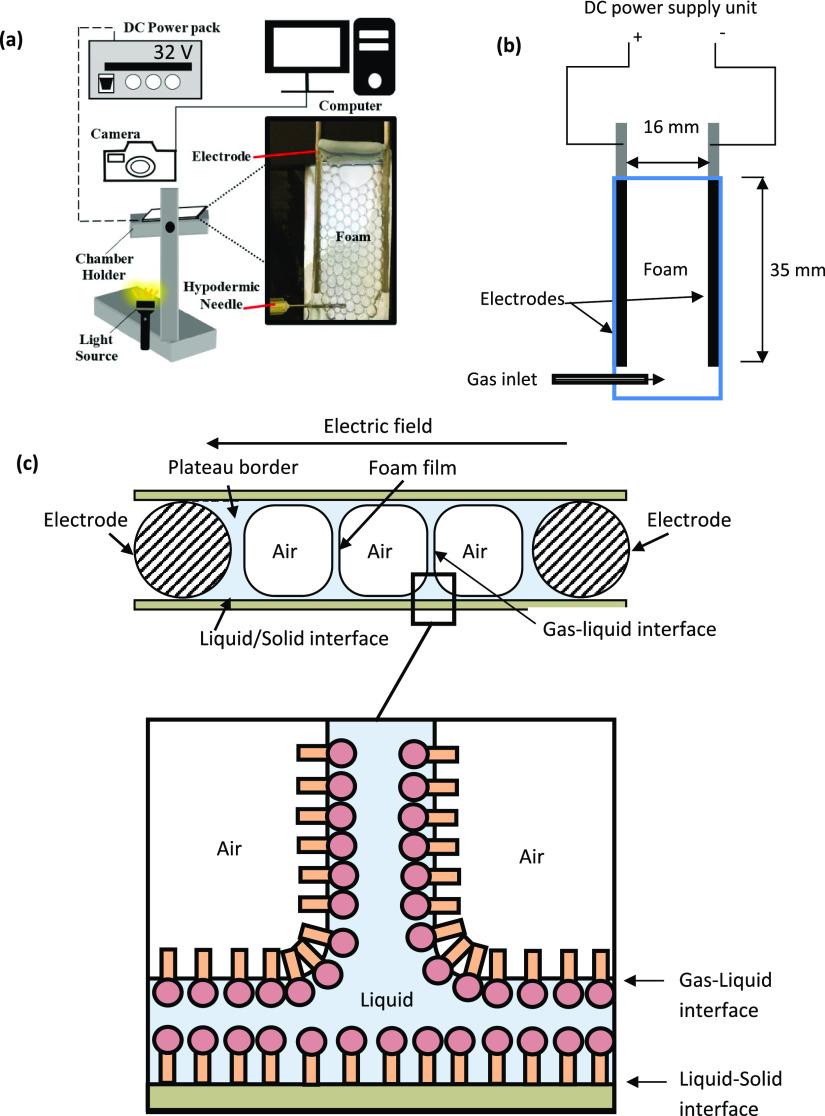
Schematic diagram of the experimental setup: (a) foam visualization setup and (b) experimental cell, plan view. Foam cell was always laid horizontally during the experiments. (c) Cross sections of the foam structure inside the chip.

A custom-made foam experimental cell was constructed by sandwiching two 35 mm long platinized titanium electrodes (ti-shop), of 2 mm diameter, between two 2 mm thick borosilicate glass slides, forming a chamber as shown in Figure 1. One side of this chamber was left open, and the opposite side was tightly sealed with epoxy resin (Devcon 5-min epoxy), leaving a small 5 mm air gap below the base of the electrodes. A hypodermic needle (internal diameter = 0.6 mm) was inserted into this gap, and that end of the cell was completely sealed again by epoxy resin to prevent any leakage. The device formed a chamber that is 2 mm deep, 35 mm long, and 16 mm wide, bounded by the electrodes and the glass slides. For two-dimensional foam generation, a predetermined fixed amount of the test solution was added into the experimental cell, and air was injected through the hypodermic needle at a fixed rate for 10 s. A supporting frame was custom-made to hold the foam cell vertically, with the air inlet positioned at the bottom of the device for foam generation. This frame could then be rotated 90°, laying the device horizontally, for operation. The device was oriented vertically during the brief foam generation stage, ensuring the creation of monodisperse foam. After foaming, the device was rotated to the horizontal orientation to negate the effects of gravity drainage on the foam sample. The initial bubble count in the whole cell was kept between 60 and 66 bubbles by visual inspection for consistency, and any experiments outside this range were discarded. For each experiment, the liquid temperature was maintained at 20 ± 2 °C. Each experiment was repeated four to six times. A second device with the same dimensions was constructed using acrylic, replacing the glass slides with acrylic slides.

### Time-Lapse Measurements

First, the device was oriented vertically, and 0.3 mL of the surfactant solution was injected into the experimental cell. Air was then bubbled through the liquid at a rate of 0.02 SLPM using a mass flow controller (Alicat MC-2SLPM-D/5 M) for 10 s to fill the chamber with foam, producing between 60 and 66 foam bubbles of nearly uniform size. The device was immediately laid horizontally, and the electrodes were attached to a DC signal generator (Thurlby Thandar PL30QMD, RS Components Ltd., Corby, U.K.) to generate an electric field across the foam. The electric field was switched on, and a camera (Logitech C270) was placed above the device to record a time-lapse video of the foam as it collapses.

Once the foam had collapsed leaving no liquid bridge between the two electrodes or 3 h had passed without a significant change in bubble count, the electric field was deactivated, and the time-lapse video was stopped. The device was removed, washed with deionized water and acetone, and dried with compressed air to prevent cross contamination of samples for the next test. This procedure was repeated by varying the surfactant solution and electric field strength between 0 and 2000 V/m. Voltages between 0 and 32 V were applied to the chip, resulting in electric fields of 0, 500, 1000, 1500, and 2000 V/m. The time-lapse videos recorded one frame per minute for the lifetime of the foam. Each frame was then analyzed using the ImageJ software (National Institutes of Health, LOCI, University of Wisconsin).

### Foam Stability Measurements

The images recorded by the time-lapse videos were analyzed to determine the number of bubbles and the bubble size distribution at any time during the experiment, allowing analysis of foam collapse with time. The percentage of bubbles remaining with respect to the initial bubble count was used as a metric of foam decay in this study, as measuring the foam height is not appropriate as this method aims to exclude the effects of gravity.

### Zeta Potential Measurements

Zeta potentials of the materials used to construct the foam cell were determined using 11 ± 2 μm borosilicate glass particles (Sigma Aldrich) and 5 μm (CV < 5%) acrylic particles (Alpha Nanotech) suspended in the four surfactant solutions used, as well as one control solution where the particles were suspended in pure water, measured using a Malvern Zetasizer 3000HS.

### Contact Angle Measurements

Contact angles were measured using a Kruss Drop Shape Analyzer 100. Drops of 3 μL of the test solutions were placed on a solid plate of either glass or acrylic, and the contact angles were determined by image analysis.

### Numerical Simulations

**Figure 2:**
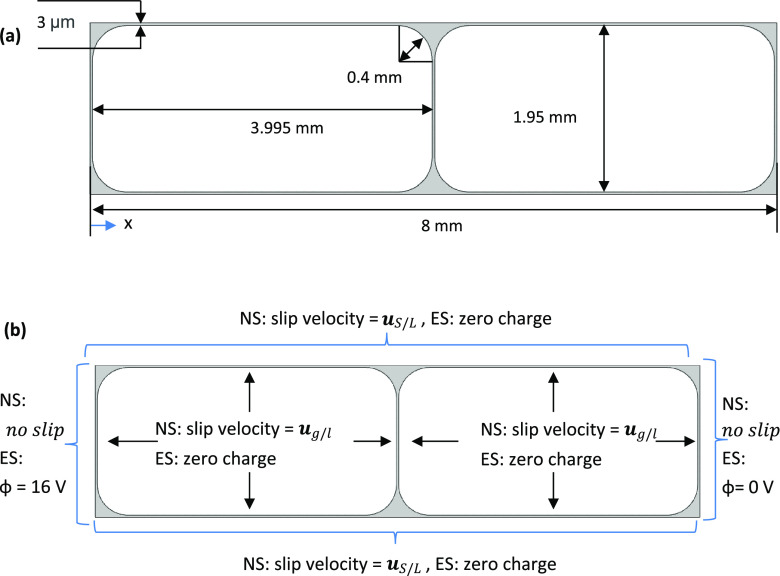
(a) Geometry for numerical simulations. (b) Boundary conditions used for numerical simulation (NS = Navier Stokes; ES = electrostatics).

Numerical simulations were performed using the finite element method (FEM) in COMSOL Multiphysics 5.5. The Navier–Stokes (NS) equations were used for fluid flow, and Laplace’s equation was used for the electric potential. The computational domain consists of a simplified 2D representation of two adjacent foam bubbles pressed against each other to simulate a region of the wet foam. Bubble shape was determined by confocal laser scanning microscopy to measure characteristic dimensions of individual bubbles in the device to produce a standard bubble model according to the geometry presented in refs (28) and (29). According to these measurements, a bubble radius of 2 mm, an inner film thickness of 3 μm, and a plateau border radius of 0.4 mm were used. The computation geometry is displayed in Figure 2a.

The Debye lengths for all test solutions were taken from appropriate experimental studies in the literature.^30−32^ The ionic strength of the test solutions and calculated Debye lengths are shown in Table 2. Temperature is assumed constant at 20 °C throughout the domain.

**Table 2:** Ionic Strength and Calculated Debye Lengths for the Test Solutions

	ionic strength (mM)	Debye length (nm)
SDS	8.2	8.81^30^
MTAB	5	5.1^31^
SB3-14	0.4	1.51^32^

The electrical double layer thickness was estimated to be in the range of 1.51 to 8.81 nm and negligible compared to the full scale of the model. The presence of the EDL was considered through slip velocity as in refs (21)(33), and (34). The electrical charge of the surfactant-covered interfaces is balanced by counterions from the bulk solution, so the net charge at the interfaces was taken to be zero. Electroneutrality condition in the bulk solution means that Laplace’s equation may be used to approximate the electric field. Under the assumption that electrical conductivity is suitably uniform in the liquid, the electric field generated within the liquid due to the applied external potential difference can be determined using Laplace’s equation:
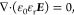
1
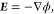
2where ε_0_ is the absolute permittivity of vacuum, ε_r_ is the relative permittivity of the liquid, *E* is the electric field, and ϕ is the electric potential. The space charge density is zero in the liquid bulk due to the electroneutrality condition outside the EDLs. Boundary conditions are defined as the electric potential at the liquid–electrode interface ϕ_*x=*0 mm_ = 16 V and ϕ_*x*=8 mm_ = 0 V, and the zero electric charge at the gas/liquid and solid/liquid interfaces (Figure 2b). As the electric charge of the surfactant-covered interfaces is balanced by the counterions, the net electric charge at these interfaces was assumed to be zero. Factoring this into gas–liquid interfaces, the normal stresses at the gas–liquid interface become zero. Therefore, any deformation of gas–liquid interfaces was neglected for the stationary model.

The governing equations for fluid flow are incompressible Navier–Stokes (Stokes’ flow as Re ≪ 1) and continuity equations for laminar flow:
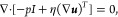
3
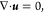
4where *p* is the pressure, ***u*** is the velocity vector, η is the dynamic viscosity, and *I* is the identity matrix. A no-slip boundary condition was set at the electrode surfaces. At the other surfactant covered interfaces, the slip velocity was determined using the Helmholtz–Smoluchowski relationship.^35^ Slip velocities at the gas–liquid (***u***_g/l_) and solid–liquid (***u***_s/l_) boundaries are given by the following:
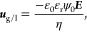
5
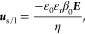
6where ψ_0_ and β_0_ are the zeta potentials at the gas–liquid and solid–liquid interfaces, respectively. The surfactants at the gas–liquid interfaces are assumed to be tangentially immobile as they are packed with surfactants above the critical micelle concentration.^36,37^ The zeta potentials are assumed to be unaffected by pH changes, as the simulation is a stationary study and does not consider the transport of chemical species over time. Moreover, pH effects are limited to the vicinity of the electrodes. The zeta potentials for all the test solutions at different interfaces are displayed in Table 3.

**Table 3:** Zeta Potential at the Gas–Liquid (from the Literature) and Solid–Liquid (Measured in This Study) Interfaces^a^

		zeta potential (mV)		
solution	type	glass–liquid	acrylic–liquid	air (gas–liquid)
SDS	anionic	–55.1	–46	–70^38^
MTAB	cationic	82	43.3	20^39^
Triton	non-ionic	–29	–11.1	–6^40^
SB3-14	zwitterionic	–37.5	–29.3	–44 (pH 9.0)^27^

a All measured at pH 7.0 unless stated otherwise.

The Galerkin finite element method (FEM) was used to solve the model in COMSOL Multiphysics 5.5. The 2D geometry was discretized using 19,198 triangular mesh elements. The number of degrees of freedom (DOFs) solved was 97,152. The computational time was approximately 69 s on an Intel Core i7 64-bit 2.60 GHz processor for each case. A mesh dependency study was carried out prior to selecting the final mesh for simulations.

## Results and Discussion

**Figure 3:**
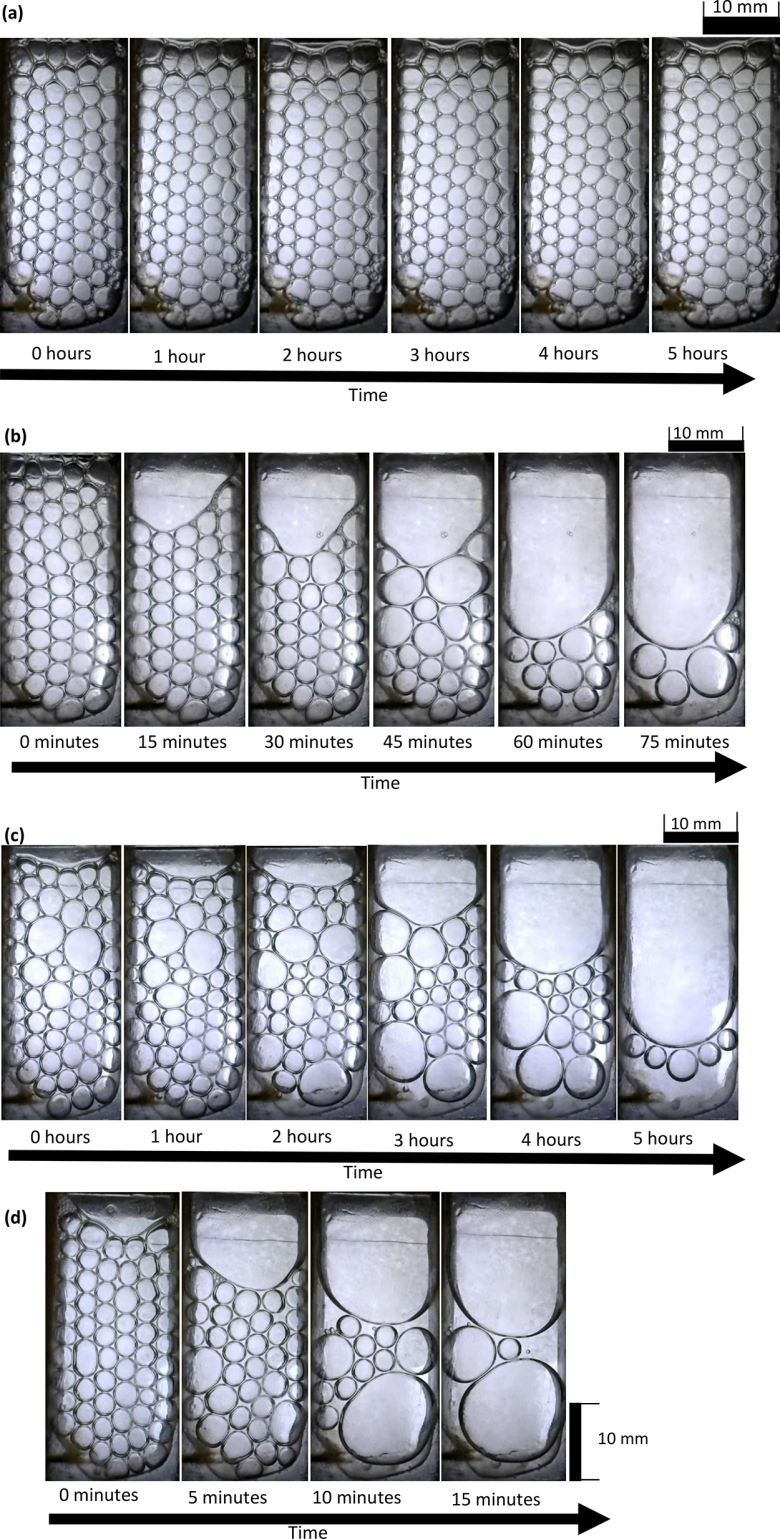
Time-lapse sequence of (a) SDS/glass, (b) MTAB/acrylic, (c) Triton X-100/glass, and (d) SB3-14/glass foam. All experiments with no electric field and horizontal chip orientation.

**Figure 4:**
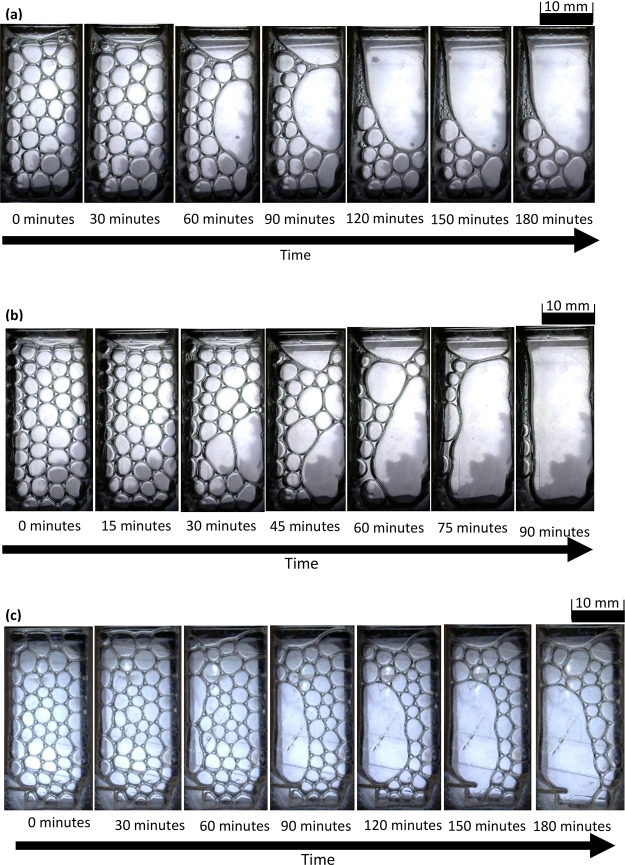
Time-lapse sequence of (a) SDS, (b) MTAB, and (c) SB3-14 foam in the acrylic device. All experiments with 1000 V/m electric field and horizontal chip orientation.

A sequence of images is presented in Figures 3 and 4 showing the collapse of foam inside the horizontally laid foam cell. The foam is generated from the gas inlet at the bottom of the images, with the electrodes situated on the left and right edges. The foam collapses over time starting from the open end at the top of the image, i.e., at the end opposite to the edge where foam was generated. During the foam generation stage, the chip was held vertically; therefore, gravity drainage could have affected the foam during this ∼10 s period leading to the removal of liquid from the upper areas of the foam. As a consequence, foam started to rupture from the top section (near the open end) even after orienting the chip horizontally. Preliminary tests attempting to generate foam in the horizontal orientation resulted in non-uniform size bubbles as the bubbles failed to separate from the nozzle immediately. When the chip was held vertically during foam generation, bubbles were immediately pulled away from the nozzle as they travel toward the top surface of the liquid. In Figure 3a, no noticeable collapse is observed, owing to the high stability of SDS foam. In Figure 3b, the MTAB foam starts to collapse from the top of the image (at the open end of the device), gradually moving downward toward the bottom of the image. In Figure 3c, the foam generated with Triton X-100 also collapsed from the top but with films appearing to rupture in the middle of the device early on, creating multiple larger bubbles that appear to consume others with time. In Figure 3d, foam made with SB3-14 collapsed relatively quickly at the top and the base of the image, resulting in the final few foam bubbles being suspended in the middle. The average bubble radii upon formation are shown in Table 4. Under an external electric field, foam collapse initiated at either electrode, gradually collapsing until the foam is only present at the other electrode, as shown in Figure 4.

**Table 4:** Average Bubble Sizes for Different Surfactant-Chip Systems

surfactant	device material	average bubble size (mm)	variance
SDS	glass	1.76	4.2%
SDS	acrylic	1.71	1.7%
MTAB	glass	1.69	3.2%
MTAB	acrylic	1.73	3.7%
Triton X-100	glass	1.75	4.1%
Triton X-100	acrylic	1.76	3.8%
SB3-14	glass	1.68	4.7%
SB3-14	acrylic	1.70	3.8%

### Foam Structure

Figure 1c presents a schematic diagram of the cross section of the device. In this configuration, the gas–liquid and liquid–solid interfaces are found at different parts of the foam structure; the gas–liquid interface is mainly situated on the lamellae between each air bubble, as opposed to the liquid–solid interfaces being mainly situated along the top and bottom surfaces of the plateau borders. As the zeta potentials at the liquid–gas and liquid–solid interfaces are dissimilar (as shown in Table 3), different flow characteristics may arise along each interface on the application of an electric field. As the two types of interfaces reside on different parts of the foam structure, the gas–liquid zeta potential will dominate in the lamellae, while the liquid–solid zeta potential will dominate in the plateau borders along the top/bottom surfaces of the foam.

### Surfactant Adsorption

Surfactant adsorption at the solid–liquid and gas–liquid interfaces must be considered before analyzing the electrokinetic flow behavior within this device. Various adsorption mechanisms may apply, depending on the charge of the surfactant and the nature of the interface.^41^ On surfaces possessing opposite charge to that of the surfactant molecules, the ionic surfactants are reported to exhibit a four-region adsorption isotherm when plotted on a log–log scale against surfactant concentration.^41^ In region I, adsorption increases linearly with surfactant concentration as the surfactant adsorption obeys Henry’s law at low concentrations.^42^ In region II, as the concentration increases, lateral interactions between surfactant molecules arise, leading to a sharp increase in the adsorption and surfactant aggregation at the interface. In region III, the adsorption rate decreases as the interfaces get saturated with surfactants, up until region IV where adsorption reaches a plateau above the CMC.

For ionic surfactants on similarly charged interfaces, electrostatic interactions do not favor adsorption as the surfactants are electrostatically repelled.^43,44^ Due to electrostatic repulsion, the adsorption of ionic surfactants onto similarly charged surfaces is related to the van der Waals and/or hydrophobic interactions between the surfactant tail and the solid surface. At lower concentrations, surfactants may adsorb vertically, where the molecule is perpendicular to the interface, with the hydrophilic head oriented toward the bulk, or if the hydrophobic interactions are strong enough to overcome electrostatic repulsion, the surfactant can adsorb laterally. As the concentration increases, surfactants increasingly adsorb vertically. This is illustrated in Supporting Information Figure S4a.

For ionic surfactants on surfaces with an opposite charge, surfactants adsorb initially by electrostatic attraction. Hydrophobic interactions between the surfactant tail and surface can cause surfactant molecules to be adsorbed both vertically, where the heads are facing the surface and tails are facing the bulk, and laterally, as the tail is attracted to the surface,^41,45^ with the vertical arrangement becoming more common as the concentration increases until the surface is completely saturated with vertically oriented surfactants.^45^ Once the surface is saturated, additional surfactants adsorb onto the monolayer through a hydrophobic chain–chain interaction, as shown in Supporting information Figure S4b, forming a surfactant bilayer,^41,46^ which corresponds to a further rise in zeta potential.^45^

The adsorption of zwitterionic surfactants is dependent on the structure of the surfactant molecule, including the length of the hydrophobic tail and the length of the spacer between head charges. SB3-14 has an intercharge carbon number of 3 and a tail carbon number of 14.^47^ Multiple adsorption mechanisms have been reported for zwitterionic surfactants. The adsorption of SB3-12 on hydrophobic solid surfaces is reported to form monolayers or hemi-cylindrical surface aggregates, as shown in Supporting information Figure S4c, and spherical surface aggregates on hydrophilic surfaces.^48^ On hydrophobic interfaces, zwitterionic surfactants preferentially adsorb with the hydrophilic head toward the bulk due to the reduction of the relatively high free energy of the hydrophobic interface upon adsorption in this manner. SB3-14 has been proposed to follow a similar adsorption mechanism.^49^ Adsorption of SB3-14 onto a solid surface will be primarily determined by van der Waals and hydrophobic interactions as opposed to electrostatic attraction due to the net neutrality of the head group.^45^ Without any surfactants, the gas–liquid interface itself is slightly negatively charged above ∼pH 4, as OH– ions adsorb at the interface.^50,51^ Surfactants adsorbed to these interfaces will alter the charge at air–water interfaces (Supporting information Figure S4d). For instance, sufficient concentrations of cationic surfactant can completely reverse the charge of the interface.^52^

Table 5 displays the measured contact angles for each surfactant solution on both acrylic and glass surfaces. Contact angles were generally higher on the acrylic surface, suggesting a more hydrophobic surface. It is worth noting that MTAB returned significantly higher contact angles on both surfaces compared to the other surfactants trialed.

**Table 5:** Contact Angles of the Test Solutions on Glass and Acrylic Surfaces

surfactant solution	contact angle on glass (°)	contact angle on acrylic (°)
SDS	15	44
MTAB	43	73
Triton X-100	15	46
SB3-14	20	42

For all surfactant solutions tested, foam stability was higher in the acrylic device compared to that of the glass device in the absence of an external electric field. The higher contact angle of acrylic compared to glass for all solutions suggests that the acrylic surface is more hydrophobic. This leads to a stronger attraction between the acrylic surface and the hydrophobic surfactant tails, leading to a stronger binding of surfactants to the surfaces that would normally adsorb with their tails toward the solid interface. Relatively weak hydrophobic interactions may explain the low stability of the SB3-14 foam inside of the glass device. The formation of surfactant bilayers on oppositely charged solids and the resulting change in zeta potential are demonstrated by the recorded zeta potential values for MTAB on glass being approximately double those of MTAB on acrylic.

Another effect of different contact angles on the two devices is that low contact angles cause liquid to spread, leading a larger plateau border area for the liquid–solid contact (see Supporting Information Figure S5); therefore, electroosmotic flow associated with solid–liquid interfaces would be spread to a larger area for the glass device compared to that of the acrylic device. The high contact angle reported for acrylic also provides greater mechanical support for the liquid lamella; hence, foam is expected to be more stable in the acrylic device compared to the glass device under no external electric fields. Whether foam stability improves or decreases within the two devices under external electric fields will depend on whether electroosmotic flow brings liquid to the lamella or removes liquid from it. The application of electric field was not found to have any noticeable effect on the contact angles at the experimental voltage range when viewed under a microscope (please see Supporting Information Figures S6 and S7).

### Factors Affecting the Foam Stability under External Electric Fields

Foam stability could be affected by various factors under external electric fields. Electroosmotic flow could potentially remove liquid from the film lamellae and transport it to plateau borders, leading to film thinning and eventually causing rupture, decreasing the foam stability. However, under certain conditions, the opposite may occur; i.e., electroosmotic flow could bring additional liquid into the film lamellae, causing them to thicken and stabilize. The film thickening behavior has been observed in free films under gravity,^11^ but further investigations would be required to verify these behaviors in the cases presented here under negligible gravity conditions. Electroosmotic velocity magnitude could differ between the gas–liquid and the solid–liquid interfaces due to different zeta potentials at these interfaces. The areas of each interface are affected by the bubble size and the contact angle, lower contact angles result in an expanded solid–liquid interface, and smaller bubble sizes mean more bubbles may be packed into the same area, increasing the surface areas of both interfaces.

The mobility of surfactant molecules in response to electroosmotic flow may also affect foam stability. Investigations carried out on cationic surfactants indicate that surfactant molecules are immobile on the gas–liquid interface under electric fields;^37^ however, this behavior has not been verified for all surfactant types. If surfactant molecules are not immobilized, inhomogeneities may be induced by surfactants moving out of place or being removed from the surface, creating surface tension gradients and inducing Marangoni stresses that may affect stability by disturbing liquid films and potentially inducing rupture.

As the electric field strength increases, the effects of joule heating will also increase. External electric fields were recently demonstrated to induce joule heating heterogeneously in liquid foam structures, resulting in the generation of thermal gradients, inducing thermal flows.^22^ This thermal Marangoni driven flow will affect liquid transport in addition to electroosmotic flow and is determined by the shape of the foam and the shape of the electric field, although the full temperature profile inside a liquid foam is yet to be calculated.^22^

The effect of an applied electric field on disjoining pressure isotherms in thin liquid films is yet to be investigated. Upon foam generation, the thickness of film lamellae inside the device is measured at around 3 μm, significantly thicker than what would be considered a common black film. From experimental studies conducted without the effect of an electric field,^53−55^ the disjoining pressure is expected to increase as film thickness decreases, with both SDS and MTAB stabilized foams appearing to rupture in the range of 10–15 nm.^53,55^ MTAB stabilized films exhibit a higher disjoining pressure in this range, implying an increased resistance to collapse at low film thicknesses. Disjoining pressure at low film thicknesses may potentially be influenced by inhomogeneities in surfactant concentration at the interface, which would affect the local surface charge. Blanc et al.^37^ suggest that the surfactant repartition of a SDS stabilized interface is not affected by the application of electric field, that such inhomogeneities do not arise, and thus that disjoining pressure may not be affected as a result. A dedicated study into the effect of an applied electric field on disjoining pressure isotherms may provide more insight on this; however, this is outside of the scope of this study.

### Foam Stability under External Electric Fields

#### Anionic Surfactant: SDS

**Figure 5:**
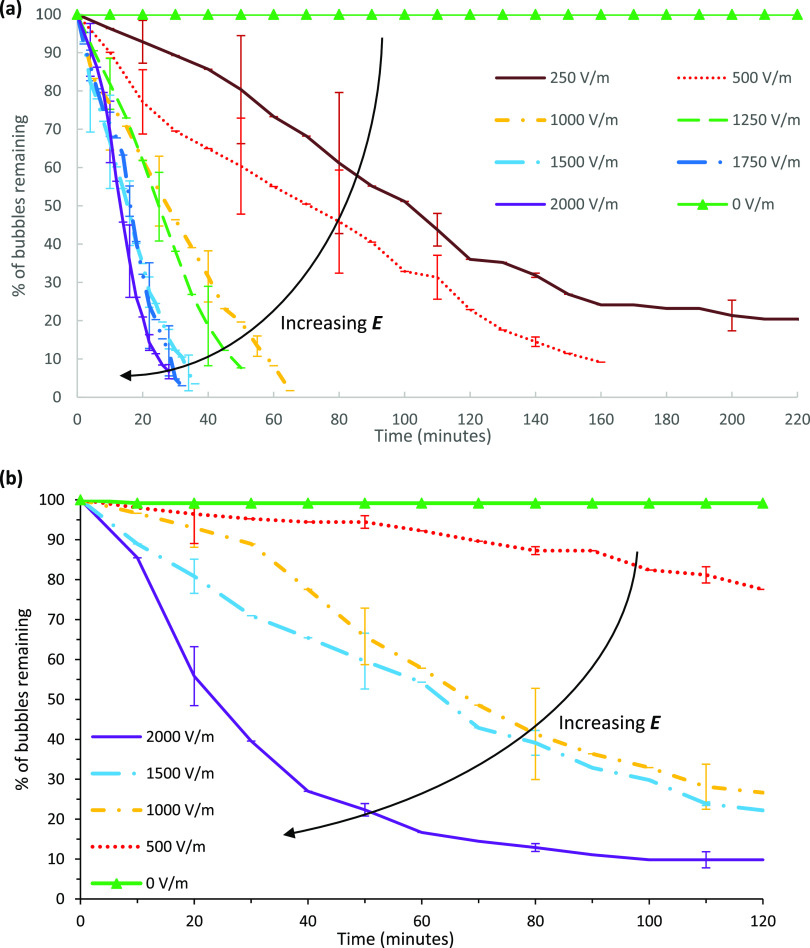
Percentage of bubbles remaining with time for electric field strengths between 0 and 2000 V/m for SDS solutions at critical micelle concentration (a) in the glass chip and (b) in the acrylic chip. Each field strength was repeated four times. Error bars represent 1 standard deviation.

Figure 5 shows foam collapse curves of SDS solutions under varying electric field strengths for the glass and acrylic devices. In both test cells, ambient stability under no electric field was high, with foams remaining stable for over 2 h without any bubble rupture. In both devices, the foam stability decreased with increasing electric field strength. In the glass chip, field strengths between 1000 and 2000 V/m exhibited rapid foam collapse, and the collapse rate appeared relatively constant, set by the electric field strength. The effects of the liquid–solid boundary are probed by changing the material of construction (solid) from glass to acrylic, which decreased the liquid–solid zeta potential, as shown in Table 3. Furthermore, it is expected that increasing the electric field strength will increase the body force exerted on the EDL, according to eq 7:^21^
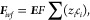
7where ***F***_*ivf*_ is the body force, ***E*** is the electric field, *c_i_* is the concentration of species *i* within the solution, and *z_i_* is the electric charge of species *i*. Stronger electric fields will induce greater electroosmotic flow, accelerating the movement of fluid close to the interfaces.

There may be several reasons for foam destabilization under external electric fields in these cases. For both test cells, increasing the electric field caused the foam to destabilize faster. First, it is possible that the removal of similarly charged and loosely bound surfactants from the films has contributed to this effect. The zeta potential at the air–liquid interface is −70 mV (at pH 7). As SDS surfactant molecules are negatively charged and the air–liquid interface itself is negatively charged at the experimental conditions,^50,51^ surfactant adsorption may be hindered by electrostatic repulsion between the interface and the charged head group. The mobility of anionic surfactants on the air–liquid interface under electroosmotic forcing has not been thoroughly investigated. If surfactant molecules can be transported from the interface by electroosmotic effects, surface tension inhomogeneities may lead to Marangoni effects, creating additional stress on the thin film, possibly affecting its stability.

Second, destabilization is likely to result from film thinning as liquid could be transported away from the films by electroosmotic flows and by thermal gradients induced by joule heating.^22^ In all cases, the foam in the acrylic chip took longer to collapse compared to that of the glass chip, and this is thought to be due to the difference between the contact angles. In both devices, electroosmotic flow on the liquid–solid interfaces is expected to be relatively weaker compared to that at the air–liquid interfaces due to the difference in zeta potentials. For instance, the time to reduce the percentage of bubbles remaining to 50% under 1500 V/m took ∼15 and 60 min for glass and acrylics devices, respectively. When each collapse curve is scaled against its time to reach 30% of the original bubble count, the curves are found to approximately fit one universal curve for each case, except for the MTAB foam. These scaled figures for SDS are included in Supporting Information Figures S2 and S3.

To understand the effects of electroosmotic flow on foam stability, lithium chloride was added to the SDS test solution at a concentration of 10^–1^ M. At salt concentrations above 1 mM, electroosmotic flow is suppressed^56,57^ due to the crowding of counterions in the electrical double layer causing ion mobility to decay.^58^ The SDS foam stability with high salt concentrations with varying electric field strengths inside of the glass device is presented in Supporting Information Figure S8. At high salinity, the stability of the SDS foam inside the glass device was diminished, reaching 50% collapse within 10 min under no electric field, compared to the stable foam for over 3 h with no noticeable decay at zero salinity. In this case, the effects of the application of an electric field did not change the foam stability noticeably, to the point where overlapping error margins make it difficult to claim any difference between the collapse curves. However, this result is not sufficient to confirm that electroosmotic flow is solely responsible for the reduction in foam stability observed in Figure 5, as time scales for foam collapse under all conditions (0–2000 V) have been significantly reduced.

#### Cationic Surfactant: MTAB

**Figure 6:**
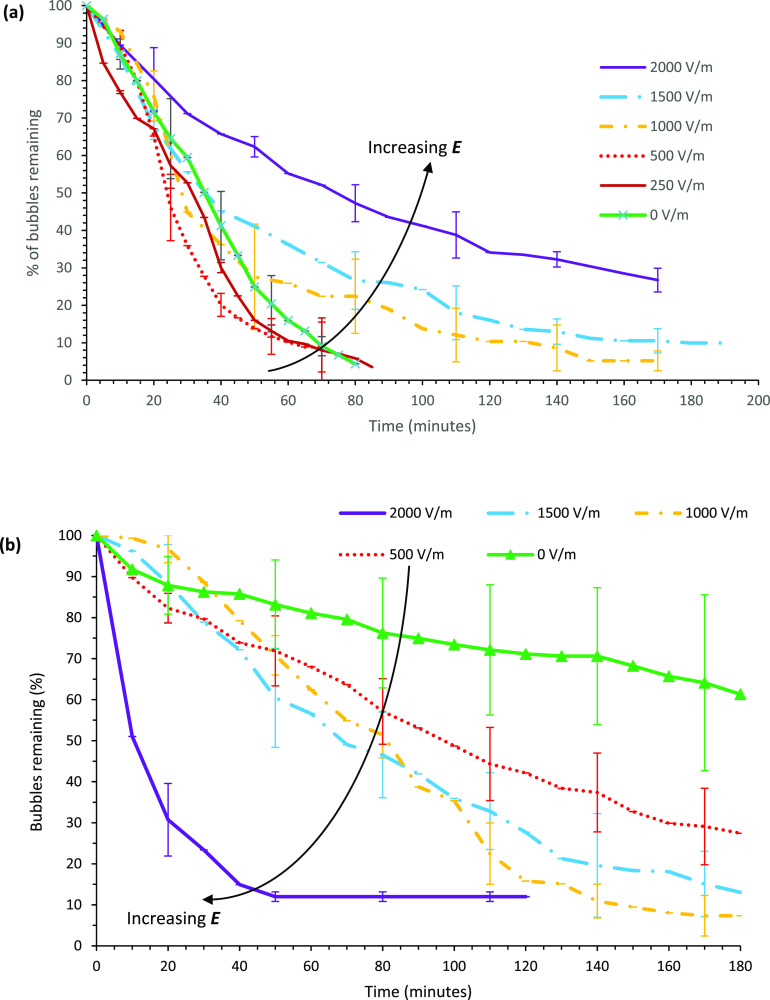
Percentage of bubbles over time for MTAB solutions between 0 and 2000 V/m in the (a) glass chip and (b) acrylic chip. Each field strength was repeated four times, and the error bars represent 1 standard deviation.

Figure 6 shows foam collapse profiles for the MTAB test solution under varying electric field strengths. In the glass chip, the stability of MTAB foam appears unaffected by the electric field strength in the range of 0–500 V/m. For 1000–1500 V/m, the stability of MTAB foam remains similar to that of the 0–500 V/m case until ∼70% of the bubbles have collapsed, and then foam stability increases with the remaining foam. For the highest electric field strength tested (2000 V/m), MTAB foam stability was noticeably increased compared to all other cases (0–1500 V) and ∼30% of the bubbles remained stable after 3 h. Sett et al.^11^ showed that both cationic DTAB films and anionic SDS films had their lifetime prolonged by the presence of electric field under no gravity. Electroosmotic velocity is dependent on zeta potential. The zeta potential of the SDS solution at the gas–liquid interface is known to be −70 mV at pH 7,^38^ and that of MTAB is estimated to be 20 mV.^59^ Hussein Sheik et al.^13,39^ previously determined the electroosmotic velocity profiles of both SDS and MTAB solutions in free liquid films using micro-PIV, concluding that the flow profiles were similar, with the direction reversed depending on the charge of the surfactant used. Under gravity, foam collapses as liquid drains downward, removing liquid from films and gradually reducing the film thickness until films are ruptured by thermal instability or other mechanical action.^60^ In the absence of significant gravity-driven drainage, electroosmotic flow is purely responsible for the flow of liquid through the thin films in our study, and so the rate of collapse is likely to be linked with the magnitude and the direction of electroosmotic flow, i.e., flow patterns near the plateau borders and lamella films. However, a different trend is observed in the acrylic chip for MTAB foam, where the foam was destabilized with the increasing electric field, as in all SDS cases.

It is interesting to note that the stability of the MTAB foam without an external electric field is significantly different between the two devices, foam being significantly less stable in the glass chip compared to that in the acrylic chip. In the glass device, the MTAB foam collapses completely in ∼80 min, whereas the MTAB foam on the acrylic device stays stable for over 3 h. This is thought to be due to the difference in contact angles, where high contact angles support liquid retention in liquid lamellas.

In the acrylic device, the stability of the MTAB foam reduced with the increasing electric field strength, as shown in Figure 6b. So why did the MTAB foam stabilize under high electric field strengths in the glass device but not in acrylic? The positive charge of MTAB molecules is expected to make strong binding with the negatively charged air–liquid interface itself. The mobility of the MTAB surfactant at the air–liquid interface was investigated in ref (37) using second harmonic generation, and it was found that MTAB molecules are immobilized at the interface and that their mobility is unaffected by electroosmotic forcing. If surfactant molecules are immobile under electroosmotic flow, then surface tension inhomogeneities and associated Marangoni effects are unlikely to cause the destabilization of MTAB foams under electric fields. However, this does not explain the opposite trend in stability in the two devices.

Now let us consider flow in the glass device. As the glass surface is negatively charged under experimental conditions, MTAB molecules may adsorb in a bilayer arrangement (Supporting Information Figure S4b), leading to a relatively high glass–liquid zeta potential of 82 mV. Furthermore, the low contact angle of the MTAB solution on glass causes the solid–liquid interface to be expanded. This combination of high zeta potential and larger area for slip velocity enhances electroosmotic flow in the vicinity of the solid surface in comparison to the air interface. Strong electroosmotic flow near the solid interface compared to that at the air interfaces could result in additional liquid being brought into the lamellae, increasing foam stability. While film thickening has been observed in response to electric field before,^11^ additional work is required to verify this behavior in 2D foams under simulated microgravity.

For the acrylic device, the solid–liquid zeta potential is roughly half that of in the glass device (43.3 mV). This corresponds to MTAB molecules adsorbing in a monolayer arrangement due to the surface being positively charged.^45^ The higher contact angle of the MTAB solution on acrylic means that the surface area for solid–liquid slip is reduced. The lower zeta potential and reduced area for electroosmotic flow at the solid–liquid surfaces mean that the overall flow will be significantly reduced in the acrylic device compared to that in the glass device. The significant difference in electroosmotic flows at the solid interface relative to the bubble interface may have resulted to this opposite behavior.

The disjoining pressure of MTAB stabilized films at 1 CMC is higher than that of SDS films at 1 CMC,^53,55^ which may result in enhanced stability when films are thinner, and potentially acts as a factor in enhanced stability under electric field. However, this does not provide a full explanation, as shown by MTAB exhibiting reduced stability under electric field in the acrylic device.

As with the SDS case, to ascertain the role of electroosmotic flow on foam stability, potassium chloride was added to the MTAB test solution at a concentration of 10^–1^ M/L to suppress electroosmotic flow. At high salinity, the stability of MTAB foam inside the glass device was diminished, reaching 50% collapse within 10 min under no electric field compared to the 30 min taken to reach 50% collapse at low salinity under no electric field (see Supporting Information Figure S9). Application of an external electric field did not produce any noticeable difference on foam stability when electroosmotic flow is suppressed by high salinity. However, since the time period for collapsing the foam under no electric field is significantly different between high and low salt concentrations, it is difficult to confirm if electrokinetic flow is the main cause of the altered foam stability under external electric fields.

#### Non-ionic: Triton X-100

**Figure 7:**
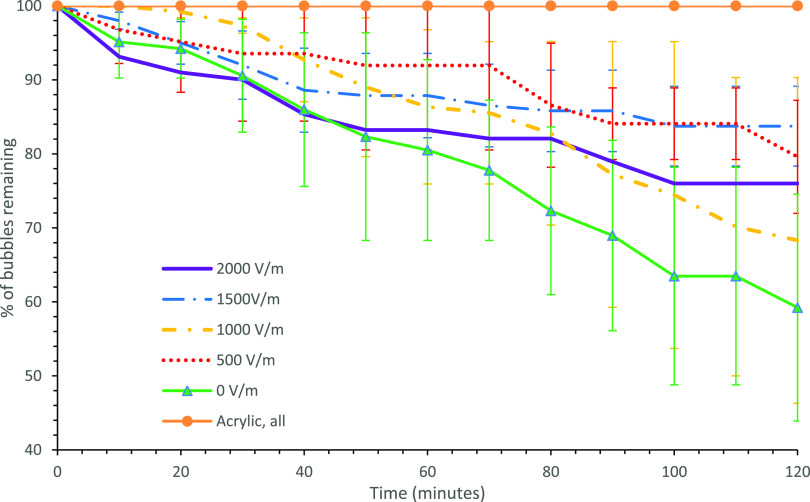
Percentage of bubbles over time for Triton X-100 solutions in the glass device. Each field strength was repeated four times, and the error bars represent 1 standard deviation.

Figure 7 shows foam collapse profiles for solutions prepared using a non-ionic surfactant, Triton X-100, under varying field strengths. The variation in stability for the non-ionic surfactant foam is not significantly affected by an external electric field, no clear trend is observed, and high error margins suggest no correlation between the foam stability and external electric field strength. Electroosmotic flows arise in systems with charged surfaces; since Triton X-100 possesses a relatively small non-zero zeta potential at glass–liquid, acrylic–liquid, and gas–liquid interfaces, as displayed in Table 3, its magnitude is significantly reduced compared to the ionic surfactants trialed. Sett et al.^11^ studied the effect of electroosmotic flow on a free liquid film stabilized by non-ionic surfactants and similarly found no effect of electric field on film stability. The foam generated inside the acrylic chip with Triton X-100 was highly stable for over 3 h with all the electric field strengths tested, i.e., no foam collapse at all. The change in stability between the two devices appears to be a result of differing surfactant adsorption mechanism onto the solid interface owing to the difference in charge and hydrophobicity between the two materials indicated by differing contact angles. This result also demonstrate that electroosmotic flow plays a key role in changing the foam stability under external fields, which is less pronounced or absent in this case due to relatively low zeta potentials at the interfaces.

#### Zwitterionic: SB3-14

**Figure 8:**
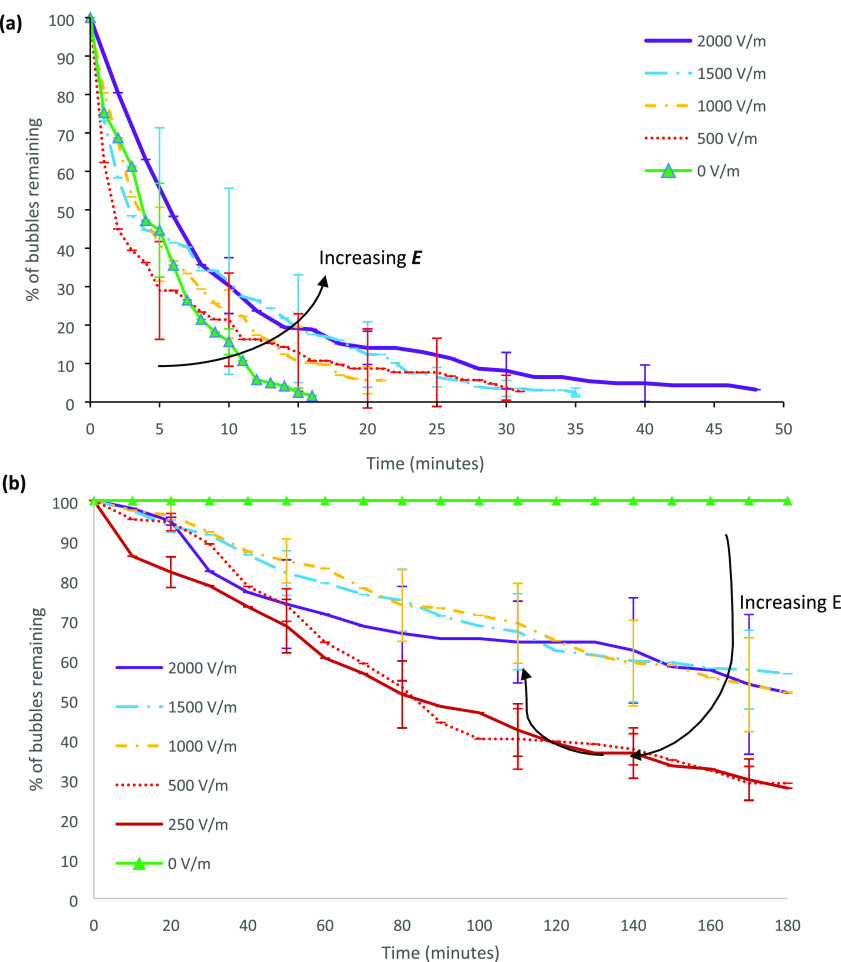
Percentage of bubbles over time for SB3-14 solutions in the (a) glass chip and (b) acrylic chip. Each field strength was repeated four times, and the error bars represent 1 standard deviation.

Foam stabilized with the zwitterionic surfactant SB3-14 was tested under external electric fields ranging between 0 and 2000 V/m. The resulting collapse curves are displayed in Figure 8. For the SB3-14 foam in the glass chip, increasing the electric field strength resulted in a marginal increase in foam stability. The time taken for the foam to reach 50% collapse increases from 5 to 7 min, and the last remaining foam bubbles took substantially longer (∼50 min) to collapse under an external electric field of 2000 V/m compared to that of the no electric field case (∼15 min). Initial collapse was fast for all field strengths, but the final few bubbles appear to be stabilized by increasing electric field strength. In the acrylic chip, the SB3-14 stabilized foam remained stable for long durations exceeding 3 h even under the maximum electric field applied. The vast change in stability between the two devices could be a result of changing the electrostatic properties of the solid–liquid interface and the adsorption kinetics of the surfactant molecules, as discussed earlier. The SB3-14 foam in the acrylic cell was destabilized at low electric field strengths tested (250–500 V/m), but further increase in the electric field (to 1000–2000 V/m) improved stability slightly compared to the low electric field cases. Overall, the SB3-14 foam stability in the acrylic chip was reduced due to the presence of an external electric field.

The time taken for 50% of the foam bubbles to collapse (τ_50%_), providing a form of half-life measurement for each case, was analyzed for cases where 50% collapse was reached within 3 h (see Supporting Information Figure S10). Only MTAB in glass and SB3-14 in glass exhibit increased half-life as electric field strength is increased. The SDS in both chips and MTAB in the acrylic chip all exhibited a decrease in half-life with increasing field strength.

### Numerical Simulation Results

**Figure 9:**
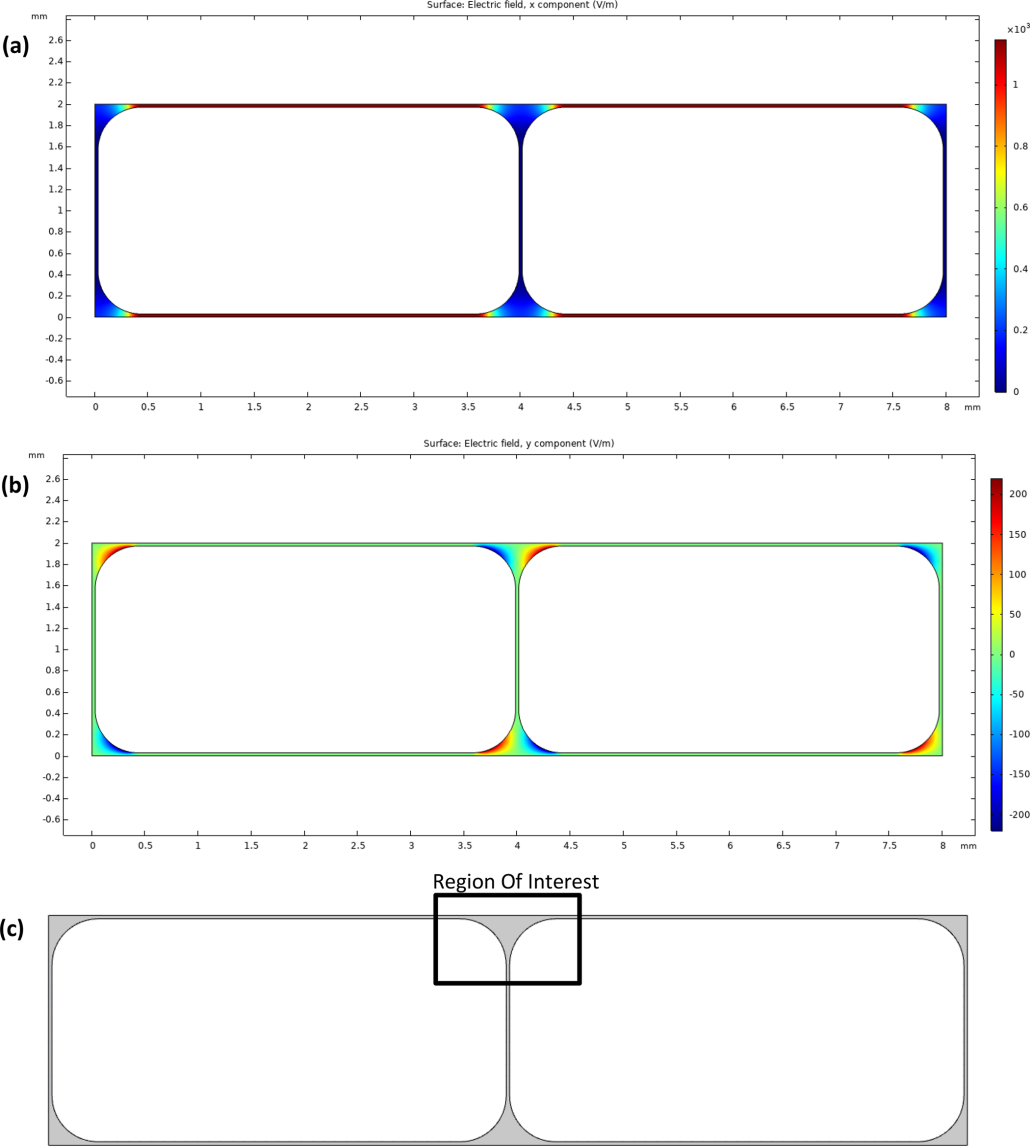
(a, b) Computational electric field (V/m): (a) ***E***_*x*_ and (b) ***E***_*y*_. (c) Illustration of the region of interest.

The velocity profiles inside the foam films were computed using Comsol Multiphysics, with particular attention to plateau borders to establish whether flow patterns in those regions support the thinnest regions of the foam (liquid lamella bridging the top and the bottom walls), where collapse is most likely to occur. Figure 9 shows the *x* and *y* components of the electric field for all surfactant cases trialed.

**Figure 10:**
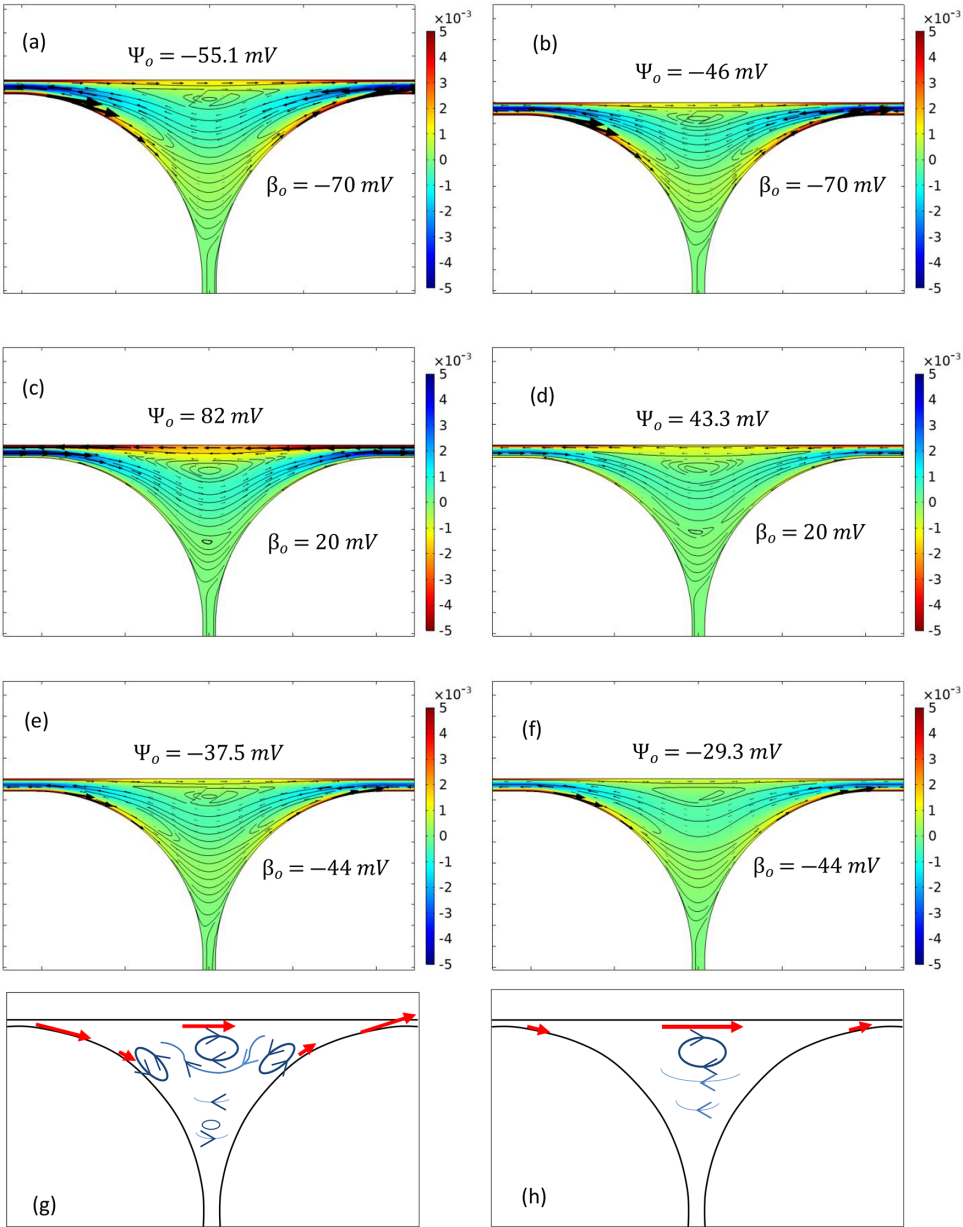
Velocity magnitude (m/s) and streamlines in the region of interest. (a) SDS foam in the glass chip, (b) SDS foam in the acrylic chip, (c) MTAB foam in the glass chip, (d) MTAB foam in the acrylic chip, (e) SB3-14 foam in the glass chip, (f) SB3-14 foam in the acrylic chip, (g) flow patterns for low ψ_0_/β_0_ ratio while both ψ_0_ and β_0_ are relatively high, and (h) flow patterns for high ψ_0_/β_0_ ratio while ψ_0_ > β_0_.

The *x* component of the electric field (***E***_*x*_, predominant part of ***E***) varies as the film curves around each bubble, intensifies along the film at the top and bottom of the simulation, and weakens around the corners of each bubble. ***E***_*x*_ is positive throughout the foam as voltage is applied on the left and right boundaries. The *y* component of the electric field (***E***_*y*_, weaker relative to ***E***_*x*_) gets distorted around the areas where the gas–liquid interfaces are not parallel or perpendicular to the electrodes, i.e., near the curved interfaces. The simulation does not take foam deformation into account and, as such, only shows a snapshot of the fluid flow as an external electric field is applied initially. Of particular interest is the region shown in Figure 9c, where the local liquid fraction is high at the corner of each bubble, where plateau borders would normally be situated in a 3D foam. The computational liquid velocity for all ionic surfactant cases trialed is displayed in Figure 10.

In general, all the flow profiles are dominated by electroosmotic flow at the gas–liquid and solid–liquid interfaces, while backflow is developed away from the interfaces. Furthermore, all the flow profiles show similar features, with the main difference being that the flow magnitude and the direction of flow depend on the surfactant used. As expected, these simulations show that the direction of flow near the interfaces is reversed when the zeta potential sign is changed from negative to positive. For instance, the MTAB foam exhibits electroosmotic flow in the opposite direction to that of SDS and SB3-14, with similar flow features observed in all cases. However, a closer inspection of the flow profiles reveals subtle differences.

For the cases shown in Figure 10a,b, the anionic surfactant (SDS) generates negatively charged gas–liquid and solid–liquid interfaces, meaning that the diffuse layer of the EDL primarily consists of positive ions.^38^ When an external electric field is applied, electroosmotic flow develops toward the cathode (i.e., left to right as in Figure 10a,b) in the vicinity of gas–liquid and liquid–solid interfaces. The high gas–liquid and solid–liquid zeta potentials for the SDS foam create a strong electroosmotic flow at both interfaces at the corner of each bubble. The domain is a closed system; therefore, to maintain continuity, a backflow is generated in the bulk, away from the interfaces, opposite to the electroosmotic flow direction (i.e., right to left in Figure 10a,b). This behavior is also observed in single film experiments.^39^ When the backflow reaches the corner of each bubble, liquid is entrained with the slip flow at the interfaces. This generates several recirculation regions within the plateau border area and may present a possible mechanism for fluid to be introduced into or removed from the lamellae, causing it to be thickened or thinned. This can be observed with streamlines in the figure. Electroosmotic flow and the resulting backflow are likely to affect the local liquid fraction, and if the local liquid fraction is reduced beyond a critical point, foam collapse will occur.^22,61^

In comparison, the MTAB foam under an external electric field shows a similar flow pattern as shown in Figure 10c,d, with the flow direction reversed due to the positive charge of the MTAB surfactant molecules. The gas–liquid zeta potential for MTAB is significantly lower than that at the solid–liquid interface, so the electroosmotic flow generated at the curved foam bubble interface (corner of each bubble) is weaker in comparison to the backflow. This suggests that there is potential for film thickening to occur, as pressure-driven backflow drives liquid down into the vertical lamellae, and the weaker electroosmotic flow at the gas interface is unable to draw liquid out of the film to counteract, leading to film thickening and foam stabilization. The simulation does not take the deformation (thinning or thickening) of the liquid films into account, so no net flow is observed in the vertical lamellae. In acrylic, the electroosmotic flow along the gas–liquid interface is relatively stronger, suggesting that it is likely to counteract backflow bringing liquid into the vertical lamellae, suppressing the stabilization effect.

For cases representing the SB3-14 foam, shown in Figure 10e,f, the gas–liquid and the solid–liquid zeta potentials are similar in magnitude, leading to a balanced electroosmotic flow and backflow within the plateau border. This may suggest that the film thickening effect of the backflow may be suppressed by electroosmotic flow, which is counterintuitive to the slight stabilization effect observed with the SB3-14 foam in the glass device. However, the stabilization observed in this case is significantly less pronounced than that observed with the MTAB surfactant on glass, and all foam stability improvements are considered relative to the highly unstable SB3-14 foam in the glass device under no electric field applied. In addition, the stabilization of SB3-14 in glass by an electric field appeared to mostly apply when the majority of the foam had already collapsed, suggesting a different mechanism to the stabilization of MTAB in glass.

It is interesting to note the slight difference in fluid recirculation regions predicted for MTAB foam in glass, where foam stability improved with increasing electric field, and all the other cases, where the foam was destabilized or stabilization was hardly noticeable. For the former case, the ratio of ψ_0_/β_0_ is relatively high (4.1) compared to the other cases (0.7–2.1, below 1 in most cases). Simulations for the non-ionic surfactant Triton X-100 were not considered in this analysis as ψ_0_ and β_0_ values are relatively low compared to other surfactant types. When ψ_0_ and β_0_ values are considerably high and the ratio of ψ_0_/β_0_ is low, three recirculation regions are developed: one above the liquid lamella near the solid–liquid boundary and two more near the gas–liquid boundary of foam bubbles on either side of the lamella. This is schematically shown in Figure 10g. Foam stability decreased with an increase in the electric field for these cases. In comparison, when ψ_0_ value and the ratio of ψ_0_/β_0_ are high, only a single recirculation region is developed near the solid–liquid boundary above the lamella, as schematically shown in Figure 10h. Foam was stabilized for these cases with increasing electric field. Even though it is hard to confirm whether these recirculation regions have a direct effect on foam stability under electric fields, it is possible that these features control the fluid flow into and out of the liquid lamella. Such stabilizing or destabilizing effects at the container boundaries should be considered when 3D foam is subjected to external electric fields. Further experimental and computational studies are required to fully understand the foam stabilization/destabilization mechanism under external electric fields.

## Conclusions

Foam stability under external electric fields was investigated by producing a monolayer of foam between two parallel plates that were placed horizontally. This configuration allowed gravity drainage to be ignored and isolated the impact of electrokinetic flow on the stability of foam produced by different types of surfactants. The material of the plates that were used to construct the foam cell had a clear effect on foam stability due to the difference in wettability (contact angles). In general, under no electric field, the acrylic cell showed higher foam stability compared to that of the borosilicate glass cell for all surfactants tested, but the SDS foam showed no collapse for over 2 h in both cells. On the application of an external electric field, the stability of the cationic foam (MTAB) in the glass cell increased noticeably at electric field strengths exceeding 1000 V/m, and the effect was stronger as foam is collapsed and a reduced percentage of foam bubbles remains in the cell. Foam produced by the zwitterionic foam (SB3-14) in the glass cell also exhibited stabilization with increasing electric field, but the effect was less pronounced. Experiments involving a non-ionic surfactant (Triton X-100) showed a relatively high experimental uncertainty and did not show a clear foam stability trend with the electric field. All other foams (SDS in the glass and acrylic cell, MTAB in the acrylic cell, and SB3-14 in the acrylic cell) collapsed faster under external electric fields compared to the respective no electric field cases. Numerical simulations performed demonstrated the importance of zeta potentials at the gas–liquid and solid–liquid interfaces for the foam stabilization or destabilization behavior. Flow patterns for all foams made of different surfactant types were predicted to be similar, but the ratio of zeta potentials at the solid–liquid interface to gas–liquid interface seemed to control liquid flow into or out of the liquid lamella, stabilizing or destabilizing foam with increasing electric field strength.

## Supporting Information Available

Chemical structures of surfactants tested (Figure S1); collapse curves of SDS and MTAB inside the glass device scaled by time to reach 30% collapse (Figures S2 and S3); illustrations of surfactant adsorption at interfaces (Figure S4); contact angle illustrations for SDS and MTAB solutions (Figure S5); contact angle observation on metal rods, with and without the electric field (Figure S6); plateau border observation near the glass surface, with and without electric field (Figure S7); collapse curves of SDS and MTAB inside the glass device at high salt concentrations (Figures S8 and S9); and foam half-lives for all cases trialed (Figure S10) (PDF)
