# Supplementary material for: Stability of Two-Dimensional Liquid Foams under Externally Applied Electric Fields
Source: Langmuir. 2022 May 12;38(20):6305–21. doi: 10.1021/acs.langmuir.2c00026 (PMC9134501; doi:10.1021/acs.langmuir.2c00026)
Supplement: Supplementary file 1 — la2c00026_si_001.pdf [file la2c00026_si_001.pdf]

Support information

Stability of two-dimensional liquid foams under externally applied electric fields

Matthieu Fauvel [a], Anna Trybala [a] Dmitri Tseluiko [b] , Victor Mikhilovich Starov [a], Himiyage Chaminda Hemaka Bandulasena [*a]

[a] Department of Chemical Engineering, Loughborough University, Loughborough, Leicestershire, LE11 3TU, United Kingdom

[b] Department of Mathematics, Loughborough University, Loughborough, Leicestershire, LE11 3TU, United Kingdom

[*]**Correspondence:** H.C.H.Bandulasena@lboro.ac.uk; Tel.: +44-1509-222515

Contents

1. **Figure S1.** Chemical structures of SDS, MTAB, Triton X-100 and SB3-14.
2. **Figure S2.** Collapse of SDS foam in glass device plotted against scaled time.
3. **Figure S3.** Collapse of SDS foam in acrylic device plotted against scaled time.
4. **Figure S4.** Surfactant adsorption for (a) ionic surfactant on similarly charged solid interface, (b) ionic surfactant on oppositely charged solid interface, (c) Zwitterionic surfactant on solid interface, (d) ionic surfactant at gas-liquid interface.
5. **Figure S5.** Schematic representation of liquid film spreading for (a): Low contact angle, (b) High contact angle.
6. **Figure S6.** *Contact angle observations for a droplet of solution of (a) SDS, (b) MTAB suspended between two electrodes with 32V applied for 10 seconds*
7. **Figure S7.** Contact angle observations inside the glass device for (a) SDS, (b) MTAB, when a voltage of 32 V is applied and relaxed.
8. **Figure S8.** Percentage of bubbles remaining with time for electric field strengths between 0-2000 V/m for SDS solutions at critical micelle concentration with LiCl concentration of 10^{-1} M in a glass chip.
9. **Figure S9.** Percentage of bubbles remaining with time for electric field strengths between 0-2000 V/m for MTAB solutions at critical micelle concentration with KCl concentration of 10^{-1} M in a glass chip.
10. **Figure S10.** Foam half-life (time for 50% of bubble collapse) of MTAB, SDS and SB3-14 solutions under varying electric field strengths.

Surfactant chemical structures

Chemical structures of Sodium Dodecyl Sulphate (SDS), Myristyltrimethylammonium bromide (MTAB), Triton X-100 and Myristyl sulfobetaine (SB3-14) are included below. SDS represents an anionic surfactant, MTAB a cationic, Triton X-100 a non-ionic and SB3-14 a zwitterionic surfactant.

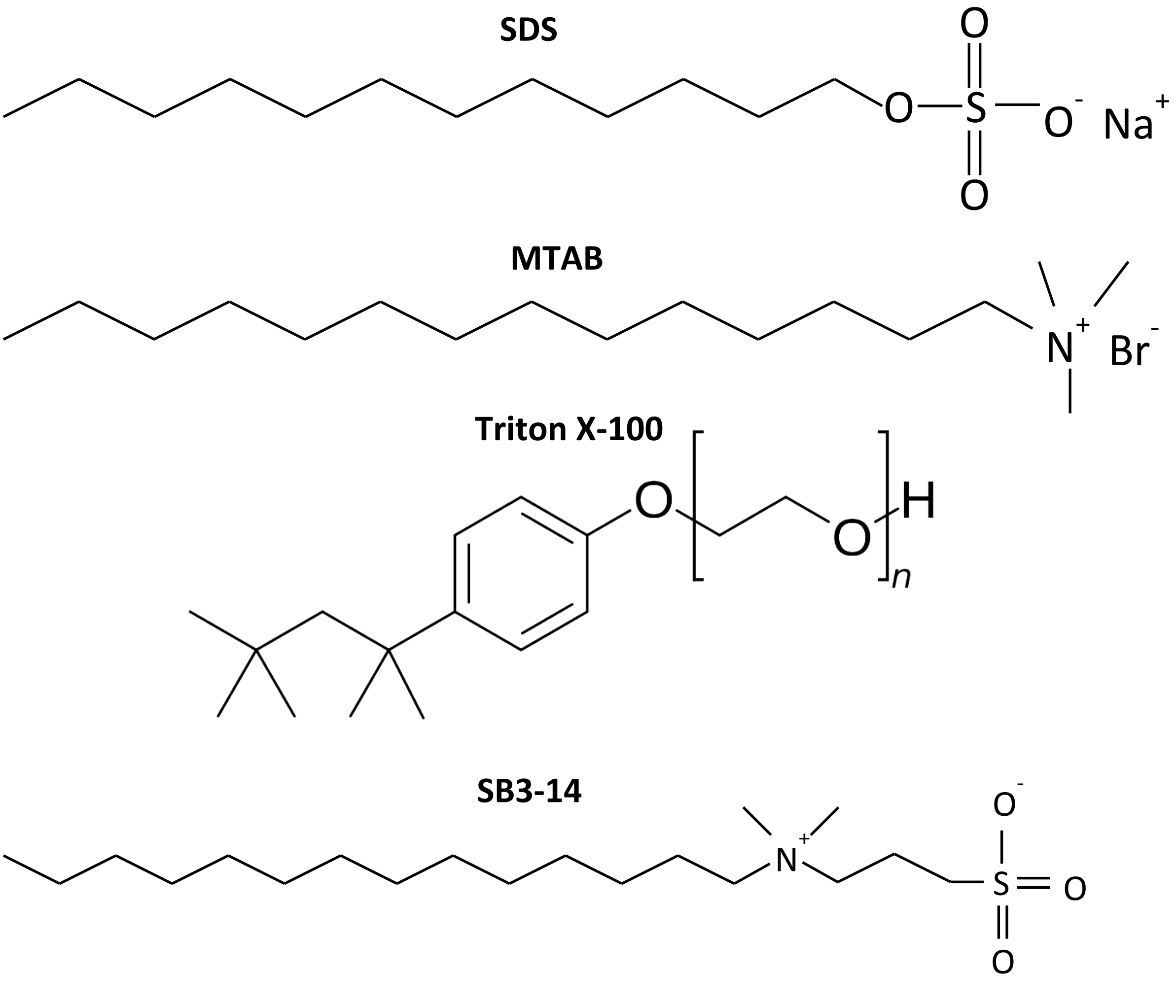

Figure S1: Chemical structures of SDS, MTAB, Triton X-100 and SB3-14

Scaled collapse curves

Foam collapse curves are plotted against a reference value $T_{30\%}$, the time taken for the bubble count in each case to reach 30% of the original value. When plotted this way all cases appear to fit a universal curve, except for MTAB. Plots for SDS are shown in Figure S2 and S3. Triton X-100 cases have been excluded as external electric fields had negligible effect on the profiles, and SB3-14 in acrylic has been excluded as only two electric field strengths reached 30% collapse.

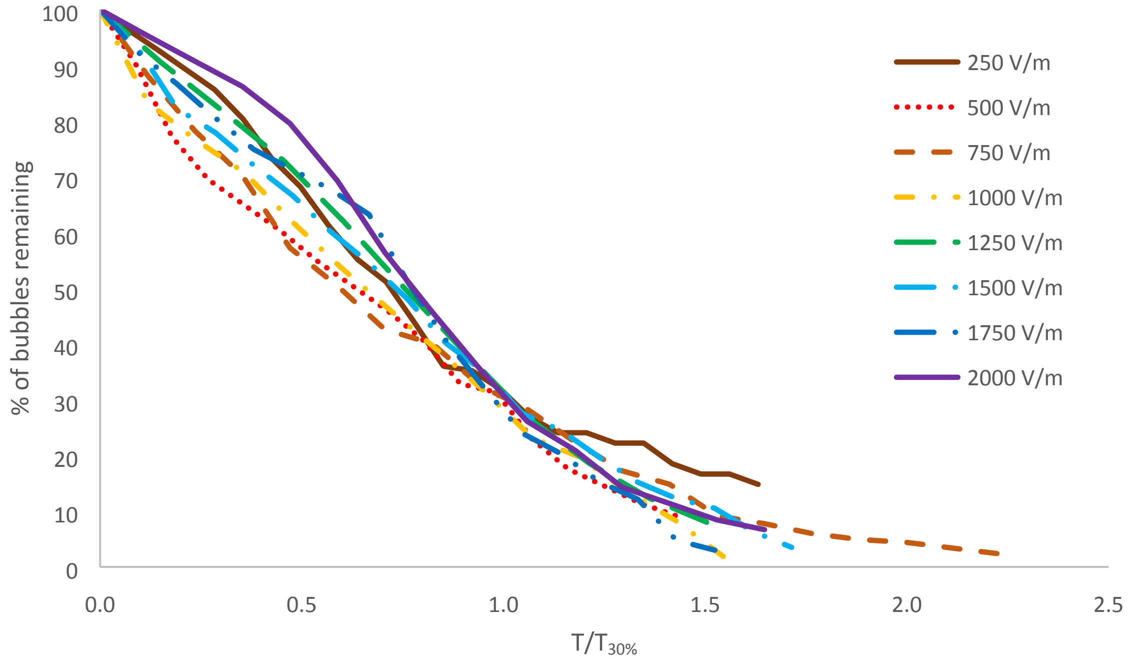

Figure S2: Collapse of SDS foam in glass device plotted against scaled time.

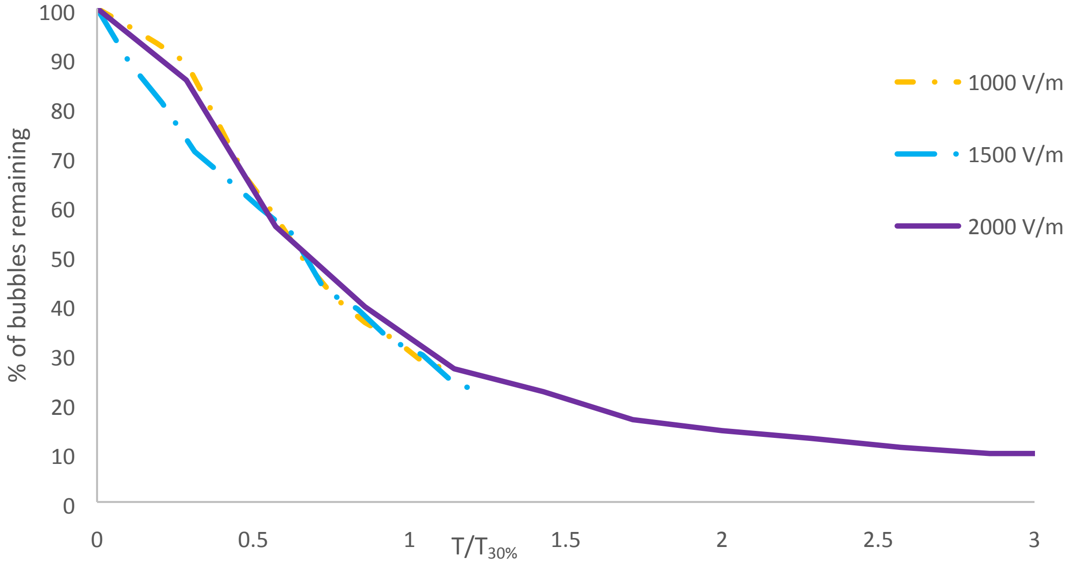

Figure S3: Collapse of SDS foam in acrylic device plotted against scaled time.

Surfactant adsorption

Figure S4 shows varying mechanisms of surfactant adsorption onto solid and gaseous interfaces. Figure S4a demonstrates anionic surfactants adsorbing onto a negatively charged solid surface, showing how ionic surfactants are arranged on similarly charged interfaces. Figure S4b shows how ionic surfactants adsorb onto oppositely charged interfaces, in this case a cationic surfactant onto a negatively charged solid surface. Figure S4c shows zwitterionic surfactants adsorbing onto a solid surface, and finally Figure S4d shows surfactants adsorbing onto a gaseous interface.

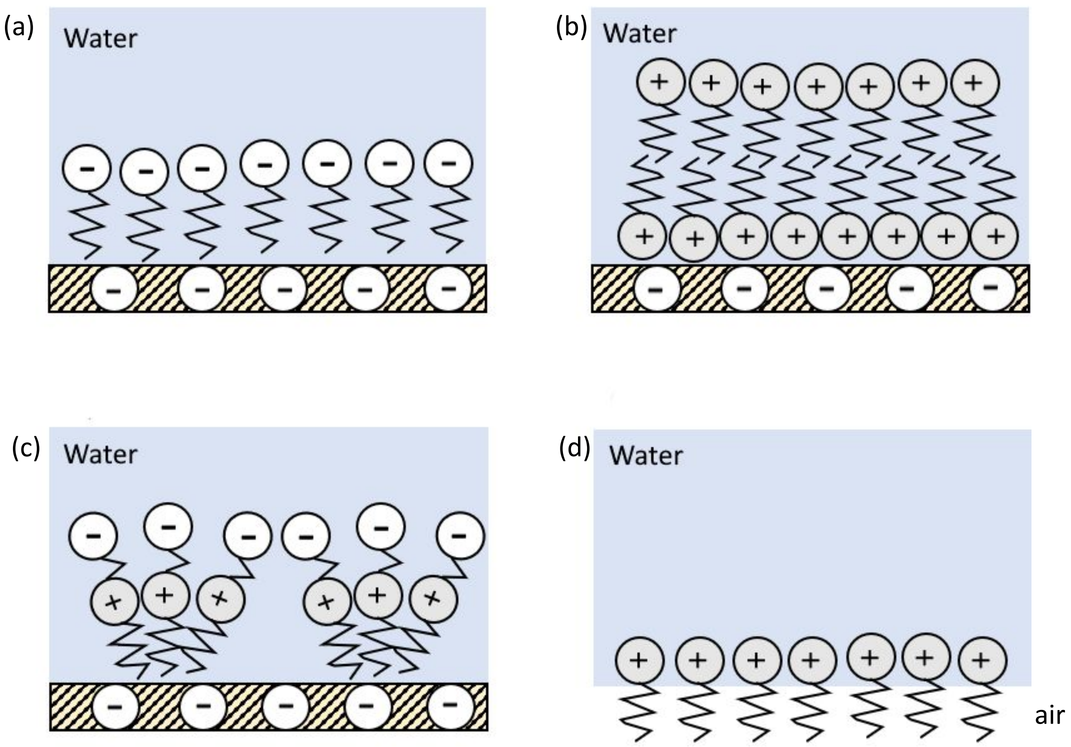

Figure S4: Surfactant adsorption for (a) ionic surfactant on similarly charged solid interface, (b) ionic surfactant on oppositely charged solid interface, (c) Zwitterionic surfactant on solid interface, (d) ionic surfactant at gas-liquid interface. Reproduced from *Khademi, M.; Wang, W.; Reitinger, W.; Barz, D. P. J. Zeta Potential of Poly(Methyl Methacrylate) (PMMA) in Contact with Aqueous Electrolyte-Surfactant Solutions.* Langmuir ***2017****,* 33 *(40).* Copyright 2017 American Chemical Society.

Contact angles

To investigate the possibility of the electric field affecting contact angles inside the device, two tests were run. The first looked at contact angles on metal surfaces, where a droplet of solution was suspended between two horizontal electrodes identical to those used in the main experimental device. A camera was placed above this to observe changes in contact angle. A voltage of 32 V was applied across the electrodes for 10 seconds and a video was recorded. Images from the video are reproduced in Figure S6.

A second experiment is carried out using the main experimental device (glass chip). Foam is generated as described in the main body, and then the device is placed under a microscope and the plateau borders are observed from above. Any changes in contact angle should incur a change in plateau border shape, visualised in Figure S5, which should be visible under microscope. A voltage of 32 V was applied across the device for 10 seconds and then relaxed, and a video was recorded of the plateau border. Images from this are reproduced in Figure S7. For both tests no observable change in contact angle was observed.

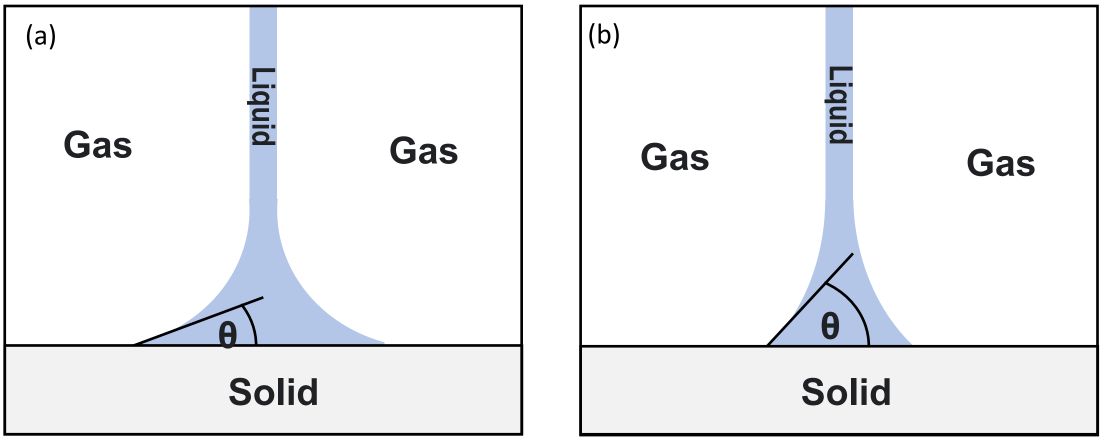

Figure *S5*: Schematic representation of liquid film spreading for (a): Low contact angle, (b) High contact angle.

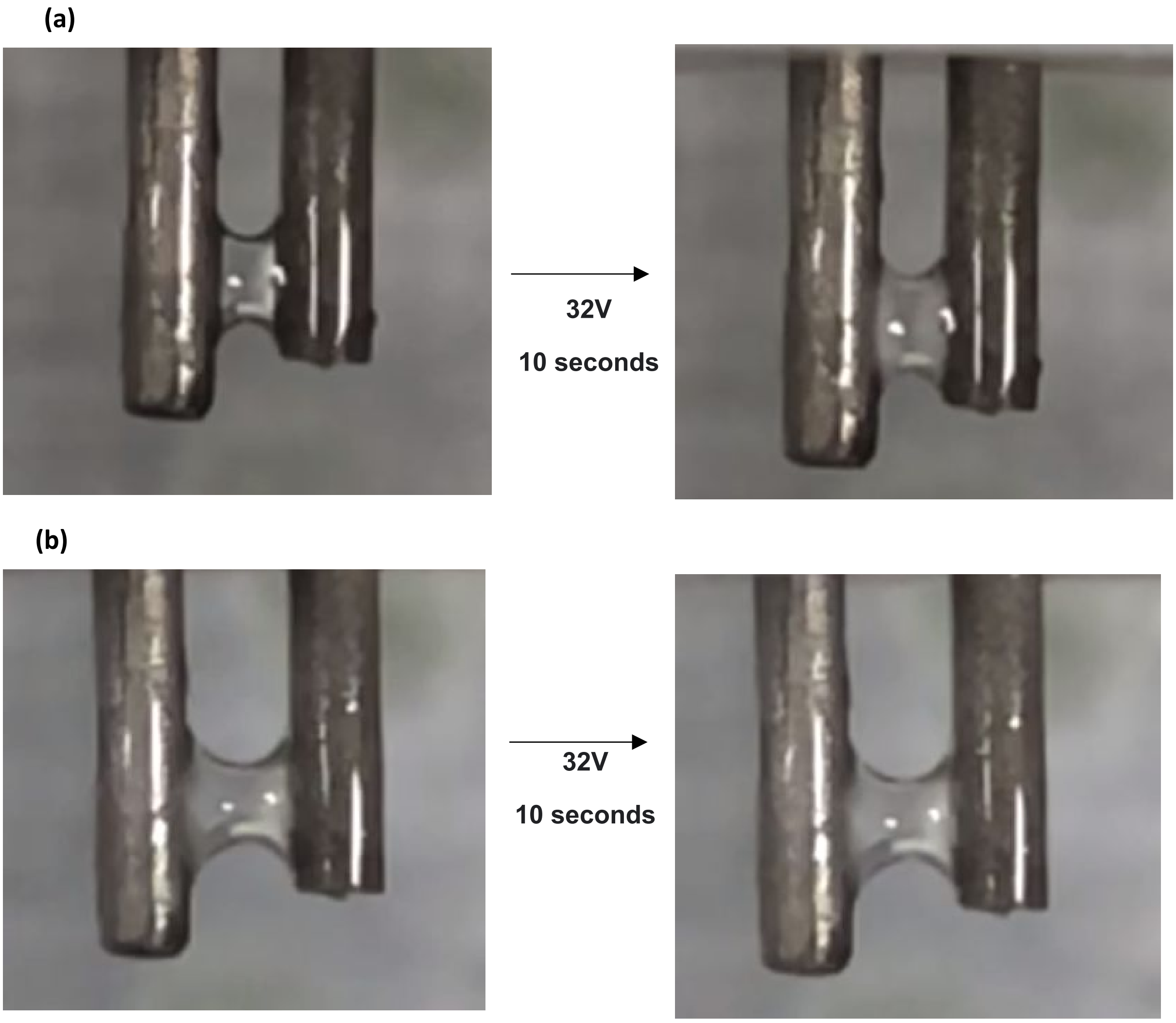

Figure S6: Contact angle observations for a droplet of solution of (a) SDS, (b) MTAB suspended between two electrodes with 32V applied for 10 seconds

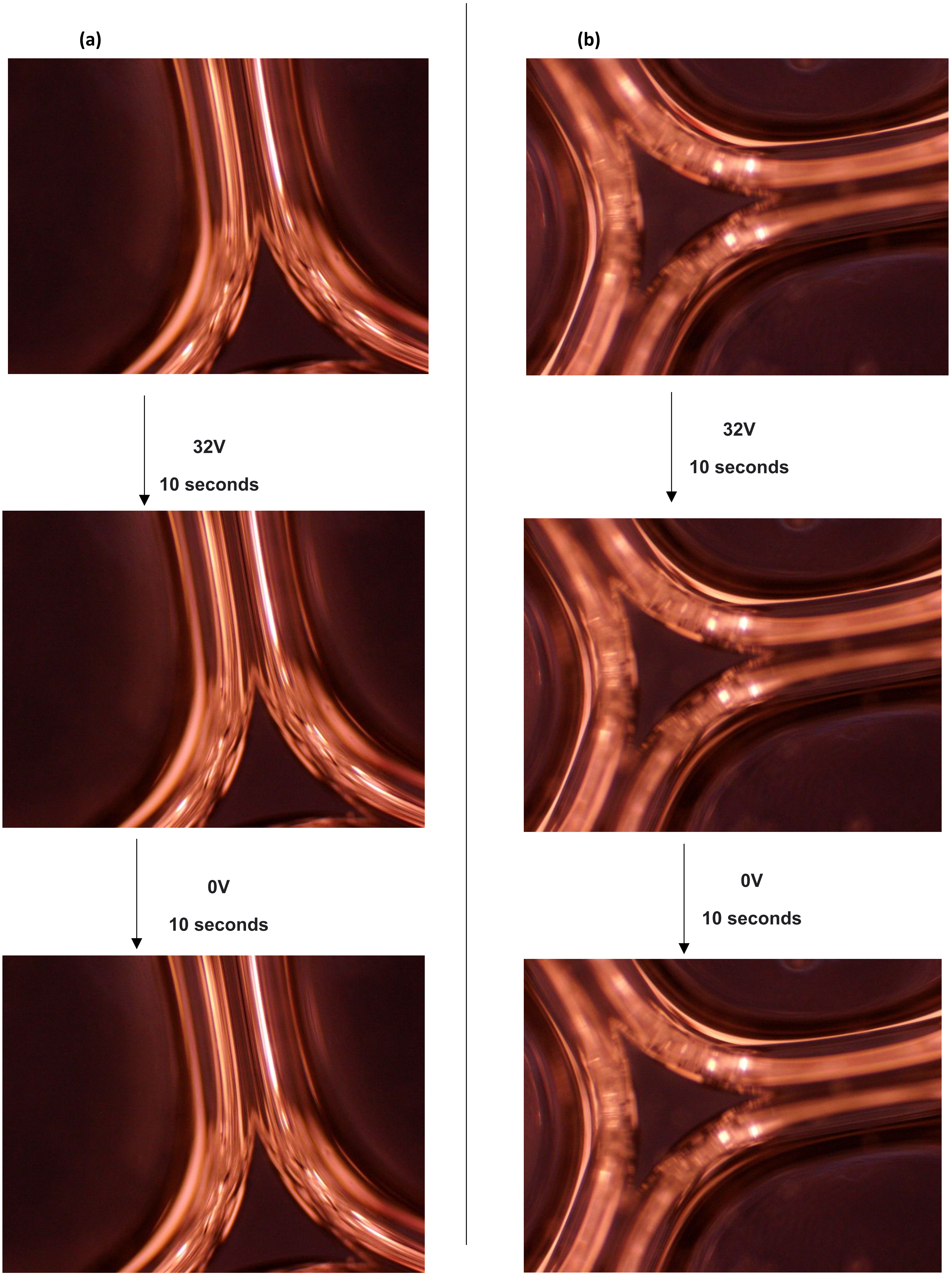

Figure S7: Contact angle observations inside the glass device for (a) SDS, (b) MTAB, when a voltage of 32 V is applied and relaxed.

Foam stability at high salt concentrations

To investigate effect of electroosmotic flow, additional experiments are run where a high concentration of salt is added to the solution to suppress electroosmotic flow. Foam collapse curves for SDS and MTAB solutions with high salt concentrations are displayed below:

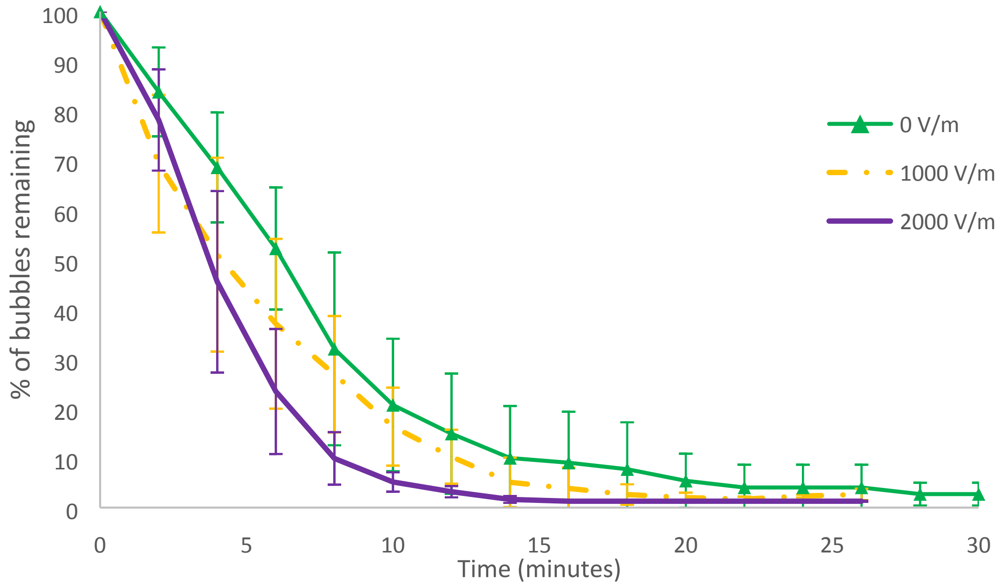

Figure S8: Percentage of bubbles remaining with time for electric field strengths between 0-2000 V/m for SDS solutions at critical micelle concentration with LiCl concentration of 10^{-1} M in a glass chip. Each field strength was repeated 4 times.

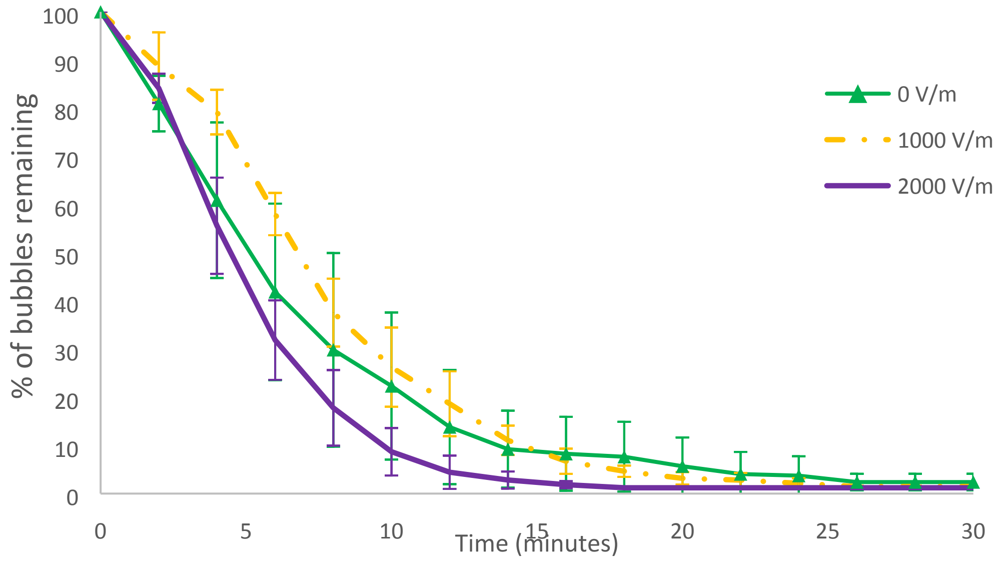

Figure S9: Percentage of bubbles remaining with time for electric field strengths between 0-2000 V/m for MTAB solutions at critical micelle concentration with KCl concentration of 10^{-1} M in a glass chip. Each field strength was repeated 4 times and the error bars represent one standard deviation.

Foam half lives

The time taken to reach 50% collapse for all cases is plotted in Figure S10. Points are only included for experiments where 50% collapse was achieved within the space of 3 hours.

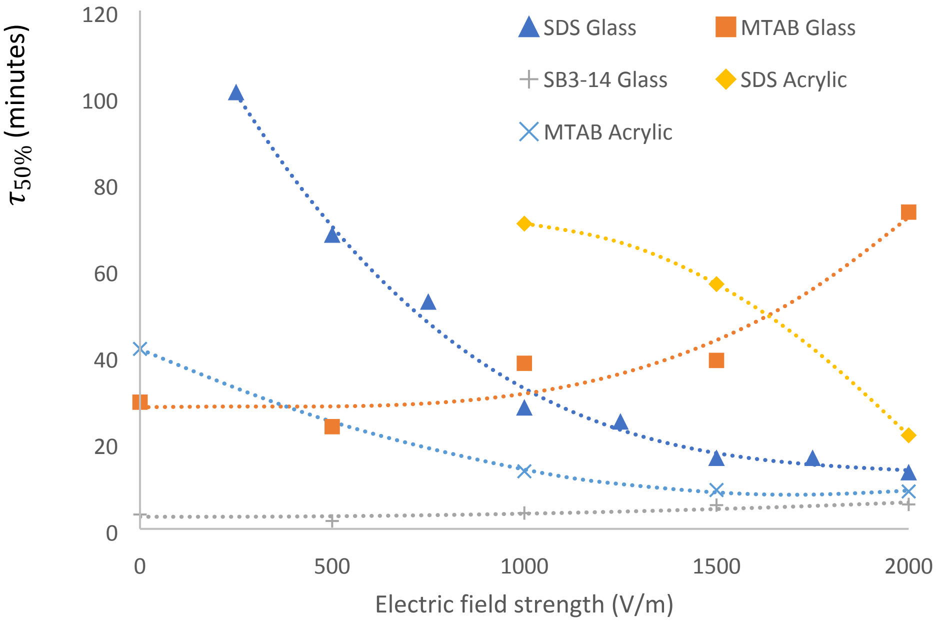

Figure S10: Foam half-life (time for 50% of bubble collapse) of MTAB, SDS and SB3-14 solutions under varying electric field strengths.
